# Influence of Plant-Derived Modifiers on the Properties of Solvent Free Epoxy Coatings

**DOI:** 10.3390/ma19153183

**Published:** 2026-07-25

**Authors:** Wojciech Żyłka, Barbara Pilch-Pitera, Piotr Potera, Renata Wojnarowska-Nowak, Małgorzata Pociask-Biały, Emilia Nowicka, Dominika Pitera, Beata Grabowska, Artur Bobrowski

**Affiliations:** 1 Faculty of Exact and Technical Sciences, Institute of Materials Engineering, University of Rzeszow, Pigonia 1, 35-310 Rzeszow, Poland; ppotera@ur.edu.pl (P.P.); rwojnarowska@ur.edu.pl (R.W.-N.); mpociask@ur.edu.pl (M.P.-B.); 2 Department of Polymers and Biopolymers, Faculty of Chemistry, Rzeszów University of Technology, al. Powstańców Warszawy 12, 35-029 Rzeszów, Poland; 3 Center of Microelectronics and Nanotechnology, Institute of Materials Science and Engineering, University of Rzeszow, al. Rejtana 16c, 35-959 Rzeszów, Poland; e.nowicka003@gmail.com; 4 Private Medical Practice Dominika Pitera, ul. Trakt Węgierski 16, 38-450 Dukla, Poland; piteradominika@gmail.com; 5 Department of Foundry Process Engineering, Faculty of Foundry Engineering, AGH University of Krakow, al. A. Mickiewicza 30, 30-059 Kraków, Poland; beata.grabowska@agh.edu.pl (B.G.); arturb@agh.edu.pl (A.B.)

**Keywords:** epoxy coatings, plant-based modifiers, UV exposure, color stability, CIELAB, roughness, gloss, Raman spectroscopy, humidity resistance

## Abstract

In this study, the effect of five natural plant modifiers, i.e., clove, ginger, dandelion, stinging nettle, and quinoa, on the surface and optical properties of epoxy coatings based on the Epidian^®^ 5/IDA system was evaluated. The modifiers were introduced in a constant amount of 3.0 wt.% of the total sample mass. The samples were subjected to short-term UV-Vis (460 nm), UVB (320 nm), and UVC (254 nm) exposure, followed by measurements of Ra/Rz roughness, CIELAB colour, gloss, Raman spectra, reflectance, light transmittance, and moisture resistance under condensation conditions. The addition of modifiers increased the roughness compared with the reference coating, particularly in the case of the coating containing dandelion (R_a_ = 2.110 µm; R_z_ = 8.463 µm in the series without UV exposure), whereas UV exposure in many cases led to partial smoothing of the surface. The greatest colour change was observed after UVB irradiation for the coating containing quinoa (ΔE* = 7.10), with a simultaneous increase in the yellow component Δb*. The 60° gloss was strongly dependent on the type of additive; the lowest values were recorded for dandelion, and relatively higher values for clove and ginger. Raman spectra confirmed the presence of bands typical of plant components, while reflectance revealed the greatest sensitivity of changes in the UV range. The condensation test after 500 h showed that plant modifiers may deteriorate the barrier properties of the coatings, particularly in the case of nettle, dandelion, and clove. The results indicate that plant modifiers are promising additives for modifying the properties of epoxy coatings, but their application requires control of particle size, dispersion, and compatibility with the matrix.

## 1. Introduction

Epoxy coatings are widely used as protective and decorative layers due to their good adhesion, chemical resistance, and the possibility of modifying their properties through the selection of functional additives [1,2]. One of the key components of such systems are pigments and fillers, which determine the appearance, hiding power, and optical stability of the coating [3,4,5,6]. In industrial practice, synthetic additives, including organic and inorganic ones, are predominant, as they provide colour intensity and appropriate coating properties [4,7,8].

However, paints production and application are increasingly being analysed in the context of environmental impact, toxicology, regulatory restrictions concerning selected hazardous substances, and the depletion of fossil raw material resources [5,9,10,11,12,13,14,15]. Many products emit toxic solvents, which is hazardous to the health of painters and users, particularly in residential interiors during renovation work. Volatile organic compounds (VOCs) emitted from coating materials constitute an important public health problem due to their widespread presence in indoor environments and their ability to induce both acute and chronic effects [16,17,18]. Inhalation exposure to VOCs may lead to irritation of the mucous membranes of the respiratory tract, neurological disorders, and aggravation of symptoms of respiratory diseases [19,20]. Particular concern is associated with compounds with documented genotoxic and carcinogenic potential, such as formaldehyde and benzene, which are present, among others, in paints, varnishes, and other coating products [16,18,19]. It has been shown that chronic exposure to formaldehyde is associated with an increased risk of nasopharyngeal cancer and leukaemia, especially myeloid leukaemia, whereas exposure to benzene is a recognised risk factor for cancers of the haematopoietic system [19,20,21]. Therefore, increasing interest is being directed towards solutions that enable the reduction in synthetic components in coating materials, including colour additives of petrochemical origin. It should be emphasised that the synthesis of some conventional pigments and additives used in the coating industry may involve the use of organic solvents and other substances that impose an environmental burden [22,23,24], which further justifies the search for more sustainable raw material alternatives.

One promising research direction is the use of plant modifiers as components of modern coating systems, allowing for the partial replacement of selected synthetic additives. In response to these challenges, increasing interest has been observed in raw materials of natural origin as alternatives to selected synthetic additives. These compounds are intensively analysed in the context of the development of more sustainable functional materials and the reduction in the use of substances classified as hazardous to health and the environment [25,26]. Current studies indicate that plant-derived materials are a rich source of naturally occurring bioactive compounds, including chlorophylls, polyphenols, flavonoids, carotenoids, tannins, and essential oil constituents. These metabolites exhibit colouring, antioxidant, antimicrobial, and UV-protective properties, making them attractive functional additives for sustainable coating systems [27,28]. In addition to imparting colour, they may modify the optical response, photostability, and mechanical properties of coating materials, thereby expanding their functionality [29,30]. Cellulose fibres and microcrystalline cellulose have been incorporated into waterborne coatings to improve mechanical strength, dimensional stability and crack resistance. Nanocellulose, including cellulose nanofibres (CNF) and cellulose nanocrystals (CNC), has attracted particular interest owing to its high aspect ratio, excellent mechanical properties and ability to enhance barrier performance, viscosity control and coating durability [31]. Similarly, lignin has been proposed as a renewable multifunctional additive providing UV shielding, antioxidant activity and partial replacement of conventional pigments or stabilisers [32,33]. Agricultural residues have also emerged as promising biofillers for coating applications. Materials such as rice husk ash, wheat straw, hemp fibres, flax fibres, coconut shell powder, bamboo fibres and cork particles have been incorporated into polymer coatings to improve hardness, abrasion resistance, thermal stability and moisture resistance while reducing the environmental footprint of coating formulations. The incorporation of finely ground agricultural biomass may additionally modify the surface texture and optical appearance of coatings, enabling the development of decorative and functional bio-based materials [34]. Plant extracts have likewise found application as functional coating additives. Curcumin, anthocyanins, chlorophyll derivatives, tannins and flavonoids have been investigated as natural pigments, antioxidants and photoresponsive compounds in protective coatings [35]. These substances can impart colour while simultaneously participating in radical scavenging processes or acting as UV-sensitive components, thereby influencing the ageing behaviour and optical response of polymer coatings. Such multifunctionality makes plant-derived additives particularly attractive for the design of sustainable smart coatings with reduced environmental impact [36,37].

Because lignin and cellulose are among the most commonly used plant biopolymers for coating modification, they are widely described in the literature. However, their effective use in paints often requires prior isolation, purification, or chemical modification to improve compatibility with the polymer matrix. These processes increase the cost and complexity of the technology. In our research, we selected dried nettle (*Urtica dioica*), ginger (*Zingiber officinale*), dandelion (*Taraxacum officinale*), cloves (*Syzygium aromaticum*), and quinoa (*Chenopodium quinoa*) for coating modification because these raw materials are characterised by a rich chemical composition and contain numerous bioactive compounds that can beneficially affect the performance of paint coatings. Furthermore, we attempted to use them in their dried and ground form, without complex purification or chemical modification processes. This makes the additive preparation process simpler, more economical, and consistent with the principles of sustainable development and the circular economy.

At the same time, direct introduction in natural form into an epoxy matrix is not a technologically simple solution. Plant particles may form agglomerates, increase roughness, reduce gloss, change colour, and disturb the continuity of the protective layer [38]. Consequently, the addition of bio-modifiers may be beneficial in terms of colour, but unfavourable from the point of view of service durability. An important factor determining the applicability of coatings modified with plant-derived bio-modifiers is light stability [39,40,41]. VIS, UVB, and UVC radiation differ in photon energy and in their ability to initiate photochemical reactions in the polymer matrix and in the additives dispersed within it. Short-wavelength UV radiation may cause fading, yellowing, binder degradation, matting, chalking, and changes in the chemical structure of the surface layer. In the case of plant modifiers, this problem is particularly important because they may exhibit limited photostability [29,30,39,40,41].

Therefore, the aim of this study was to obtain solvent-free epoxy coatings modified with plant additives and to evaluate their properties in terms of application as paints that are safe for health. For this purpose, it was assessed how the type of plant-derived modifier affects the structure and properties of the coatings. To this end, exposure tests to UV-Vis, UVB, and UVC radiation were performed, and the stability of the coatings was evaluated using roughness, gloss, and colour measurements, Raman spectroscopy, UV-Vis-NIR spectroscopy, and a solar radiation simulator.

## 2. Materials and Methods

### 2.1. Materials and Coating Preparation

Epidian^®^ 5 epoxy resin and isophorone diamine hardener (PDA) were supplied by Sarzyna Chemicals (Nowa Sarzyna, Poland). Stinging nettle (*Urtica dioica*), quinoa (*Chenopodium quinoa*), and dandelion flowers (*Taraxacum officinale*) were harvested from a local farm in June 2025 and dried (in Rzeszów, Poland). Clove, in the form of dried clove buds (*Syzygium aromaticum*), was purchased from a local supplier (in Rzeszów, Poland); the country of origin was Madagascar.

Ginger, in the form of fresh ginger rhizomes (*Zingiber officinale*), was purchased from Jeronimo Martins (Lisbon, Portugal); the country of origin was China. Fresh ginger rhizomes were sliced and dried at 60 °C for 48 h until constant mass was reached. The dried material was then ground and sieved through a 100 µm sieve before incorporation into the epoxy resin. The remaining plant modifiers were also finely ground and sieved through a 100 µm sieve to obtain a uniform particle size fraction for paint formulation. A reference sample without modifier and five compositions containing plant modifiers were prepared. The plant-based modifiers were thoroughly dispersed in Epidian^®^ 5 epoxy resin. The resin-to-hardener weight ratio was 2:1, and the modifier content relative to the total sample mass was approximately 3.0 wt.%. The composition of the coatings is presented in Table 1.

**Figure 1:**
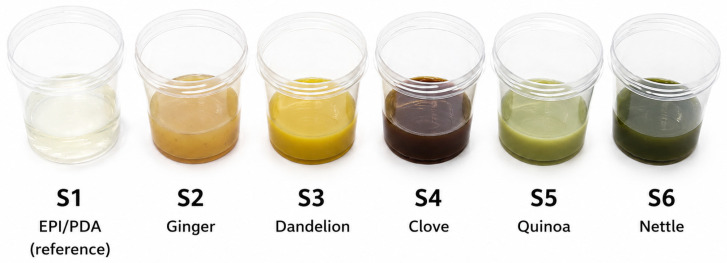
Prepared samples of solvent-free epoxy paints containing natural modifiers.

Immediately before application to the substrate, the samples were mixed with the IDA hardener for approximately 2–3 min until the most homogeneous mixture possible was obtained (Figure 1).

The mixture was applied to Q-panel R-36 (Q-lab, Westlake, OH, USA) substrates using a standard applicator bar (gap thickness: 200 µm; Research and Production Laboratory of Rzeszow University of Technology, Rzeszow, Poland), ensuring the formation of a uniform coating layer for further testing. In addition, samples were prepared on a PTFE substrate, which enabled easy separation of the cured coating. The samples were cured at room temperature (20–25 °C) for 24 h and then conditioned for 7 days.

### 2.2. UV Exposure

After curing, the samples were irradiated using lamps emitting UV-VIS, UVB, and UVC radiation (Table 2). UV-VIS exposure was carried out using a device emitting radiation with a wavelength of approximately 460 nm for 15 min (Research and Production Laboratory of Rzeszow University of Technology, Rzeszow, Poland). UVB exposure was performed using an EMITA VP-60 lamp (Fabryka Aparatury Elektromedycznej Famed-1, Łódź, Poland) with a wavelength of approximately 320 nm for 15 min. UVC exposure was carried out using an EPROM Eraser device (Shaoxing Qiaoda Technology Factory, Shaoxing, China), emitting radiation with a wavelength of approximately 254 nm, also for 15 min. The applied conditions made it possible to compare the response of the coatings to short-term exposure to three radiation ranges while maintaining the same exposure time.

### 2.3. Characterisation Methods

The surface topography was evaluated using a MarSurf PS1 profilometer (Mahr, Göttingen, Germany), determining the R_a_ and R_z_ parameters in accordance with PN-EN ISO 21920-2 [42]. For each sample, three measurements were performed, and the results are presented as the mean ± standard deviation. Colour was measured using a Test-An 45/0 DT-800 colorimeter (Anticorr, Gdańsk, Poland) in the CIELAB colour space, and the colour difference ΔE* was calculated relative to the reference sample from the same exposure series. Gloss was determined using a micro-TRI-gloss µ gloss meter (BYK-Gardner, Geretsried, Germany) at geometries of 20°, 60°, and 85° in accordance with PN-EN ISO 2813 [43], with the 60° angle used primarily in the main analysis. Raman spectra were recorded using a Renishaw microspectrometer with a 785 nm laser, 5% power, an exposure time of 10 s, and three accumulations (Renishaw, Wotton-under-Edge, UK). Spectral reflectance R(λ) was measured using an Agilent Cary 5000 UV-Vis-NIR spectrophotometer in the range of 200–2500 nm (Agilent Technologies, Santa Clara, CA, USA). Light transmission was estimated using a QuickSun 130CA solar simulator (Endeas Oy, Oulu, Finland), based on the short-circuit current I_SC_. The resistance of the coatings to moisture was assessed in accordance with PN-EN ISO 6270-2 [44] (condensation conditions) in a TestAn 220 humidity chamber (Anticorr, Gdańsk, Poland). Samples coated on steel Q-panels R-36 (Q-lab, Westlake, OH, USA) were placed on grids in the chamber’s working space. Conditions corresponding to a high-humidity environment were set, favoring the condensation of water vapor and the retention of condensation on the surface:

- temperature: 40 ± 3 °C,
- humidity: ~100% RH (condensation conditions),
- exposure time: 500 h, continuous mode.

After the exposure, a visual assessment of the coating condition was performed, focusing on changes such as discoloration, cracking, delamination, and subcoating corrosion according to PN-EN ISO 4628 [45]. The optical microscope LAB 40 (Opta-Tech, Warsaw, Poland) was used to initially assess the surface morphology of the coatings. Adhesion to the substrate was evaluated using the cross-cut test according to PN-EN ISO 2409 [46]. The coating was incised with a TestAn 535 Automatic Cross Hatch Tester (Anticorr, Gdańsk, Poland). The cutting parameters were as follows: landscape cuttings: 6, portrait cuttings: 6, intervals: 2.00 mm, load: 15 N, speed: 5 mm/s, angle: 90°. Adhesion quality was evaluated visually on a six-point scale, where 0 indicates excellent adhesion (smooth incision edges with no coating loss) and 5 indicates poor adhesion (surface damage exceeding 65% of the grid area). Relative hardness was measured using a König pendulum hardness tester (BYK-Gardner, Geretsried, Germany) in accordance with PN-EN ISO 1522 [47]. A glass plate (176 oscillations) was used as a benchmark representing the maximum attainable surface hardness. Scratch resistance was tested using a manual Clemen scratch tester (Elcometer, Nieuwegein, The Netherlands) in accordance with PN-EN ISO 1518-1 [48]. The static and dynamic friction coefficient was determined by the horizontal plane method (Guang Zhou BiaoJi Packaging Equipment Co., Ltd., Guanghou, China) according to PN-EN ISO 8295 [49].

## 3. Results and Discussion

Plant additives were selected for the study and used in their natural form after grinding and drying, namely stinging nettle (*Urtica dioica*), quinoa (*Chenopodium quinoa*), dandelion flower (*Taraxacum officinale*), clove (*Syzygium aromaticum*), and ginger (*Zingiber officinale*), in order to investigate their effect on the properties of the coatings. The selected additives are commonly cultivated plants used as herbs or spices and are known for their beneficial effects on humans. In addition, the low-molecular-weight epoxy resin Epidian^®^ 5 was used. The resin is liquid at room temperature, allowing the modifiers to be effectively dispersed without the use of additional solvents. Consequently, the coating system does not release volatile organic solvents during either application or curing, which constitutes a significant advantage of the developed formulation.

### 3.1. Surface Roughness

**Figure 2:**
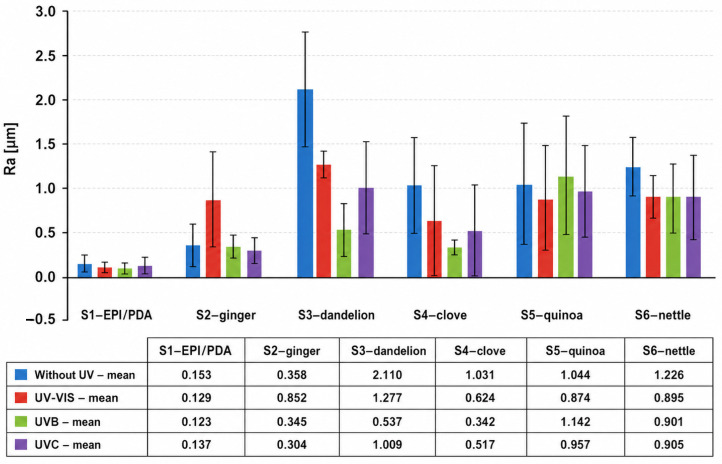
Comparison of mean R_a_ values [µm].

**Figure 3:**
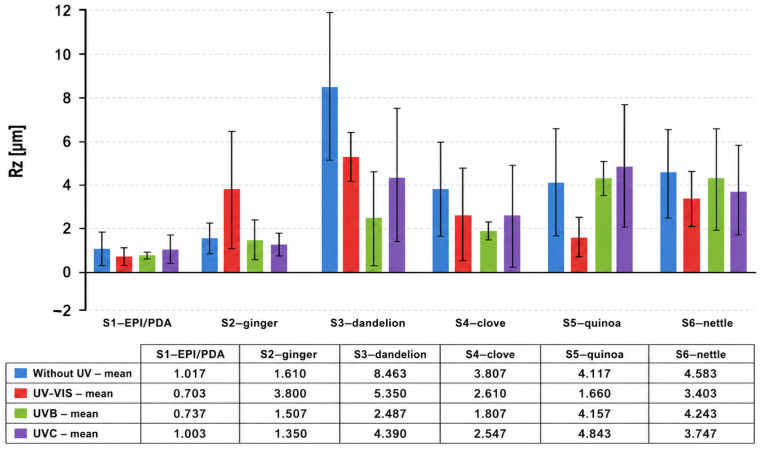
Comparison of mean R_z_ values [µm].

The values of the R_a_ and R_z_ parameters are summarised in Table 3, while their graphical comparison is presented in Figure 2 and Figure 3. The reference coating without modifiers was characterised by the lowest roughness in the entire set of samples. In the series not exposed to UV radiation, the values were R_a_ = 0.153 µm and R_z_ = 1.017 µm. The introduction of plant powders increased the roughness, indicating the formation of a more heterogeneous surface layer. The strongest effect was observed for dandelion, for which R_a_ reached 2.110 µm and R_z_ reached 8.463 µm. Among the modified samples in the initial state, ginger exhibited the lowest roughness. Greater roughness values for coatings containing plant particles may result from their different chemical structure, which affects the change in interactions with the epoxy matrix. This can result in varying additive wettability and compatibility with the resin matrix. The better the additive’s compatibility with the resin, the easier the additive disperses and the smoother the coating.

Based on the obtained results, it can be stated that the behaviour of the coating depends not only on the energy of UV radiation, but also on the type of modifier. In most systems, UV exposure led to a decrease in roughness compared with the non-irradiated samples, particularly in the case of dandelion and clove. This may be associated with degradation of the surface layer, flattening, and crumbling of local modifier agglomerates. However, this trend was not observed for all samples: the coating containing ginger showed an increase in R_a_ after UV-Vis exposure, whereas the coating containing quinoa reached the highest R_z_ value among all the tested samples after UVC irradiation. This may be due to the lower resistance of ginger and quinoa to radiation than the other modifiers or their weaker interaction with the epoxy matrix, which resulted in the crumbling of local agglomerates and the formation of cavities in the coating [36].

### 3.2. Color Stability After UV Exposure

**Figure 4:**
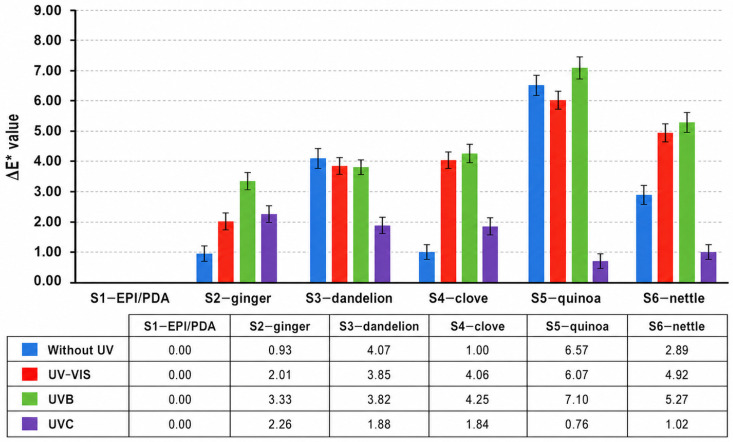
Plot of mean colour differences ΔE*.

**Figure 5:**
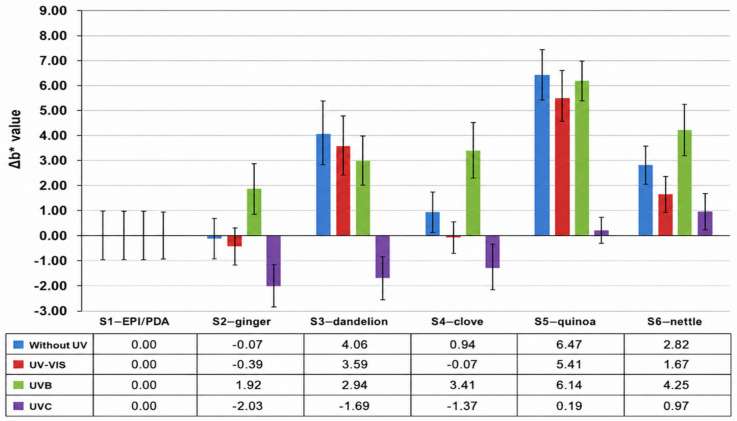
Plot of changes in the colour component Δb*.

Colour analysis in the CIELAB colour space showed that plant modifiers have a significant effect on the colour of epoxy coatings. In the series without UV exposure, the greatest colour difference relative to the reference sample was observed for the sample containing quinoa (ΔE* = 6.57) and dandelion (ΔE* = 4.07). This change resulted mainly from an increase in the b* component, indicating an increased contribution of yellow colour. These modifiers are suitable as colouring additives for coatings with natural, pastel colours. The coating containing ginger and clove showed a much smaller colour difference compared with the reference sample, with ΔE* values below or close to 1, which is not noticeable to the naked eye. This means that these additives may rather act as fillers in coatings, having only a minor effect on colour change. The corresponding numerical values are summarised in Table 4, and their graphical comparison is presented in Figure 4 and Figure 5.

The greatest colour change after UV exposure occurred after UVB irradiation, especially in the case of quinoa (ΔE* = 7.10) and nettle (ΔE* = 5.27).

UV-VIS radiation also caused noticeable colour changes in the samples containing quinoa and nettle, but these changes were smaller than those observed after UVB irradiation. After UVC exposure, the ΔE* values were relatively lower (0.76–2.26).

The obtained results indicate that UVB radiation has the greatest impact on the colour stability of epoxy coatings modified with plant-based additives. Therefore, these coatings should not be used in applications involving prolonged exposure to outdoor conditions, particularly to UVB radiation. However, this does not represent a major limitation of the proposed system, as epoxy resins are inherently susceptible to photodegradation under UV exposure and are generally not intended for long-term outdoor applications due to their limited weathering resistance.

### 3.3. Gloss and Visual Appearance

**Figure 6:**
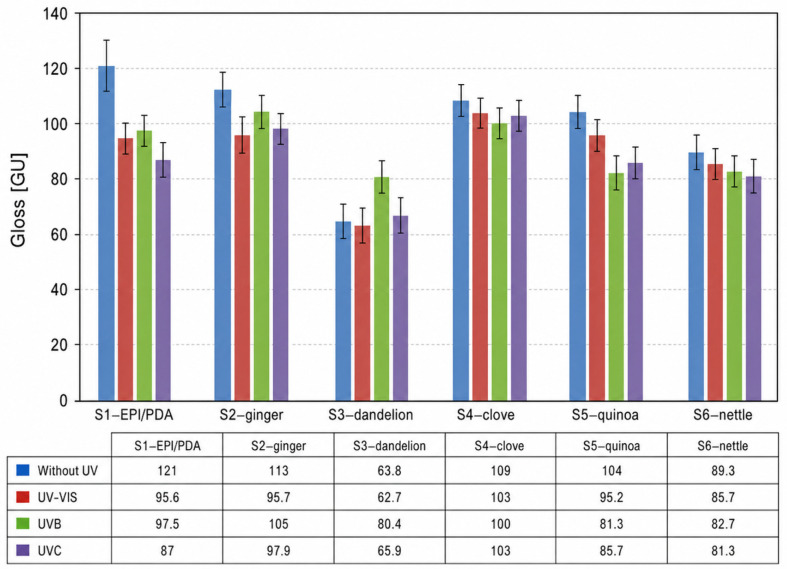
Plot comparing 60° gloss values [GU].

**Figure 7:**
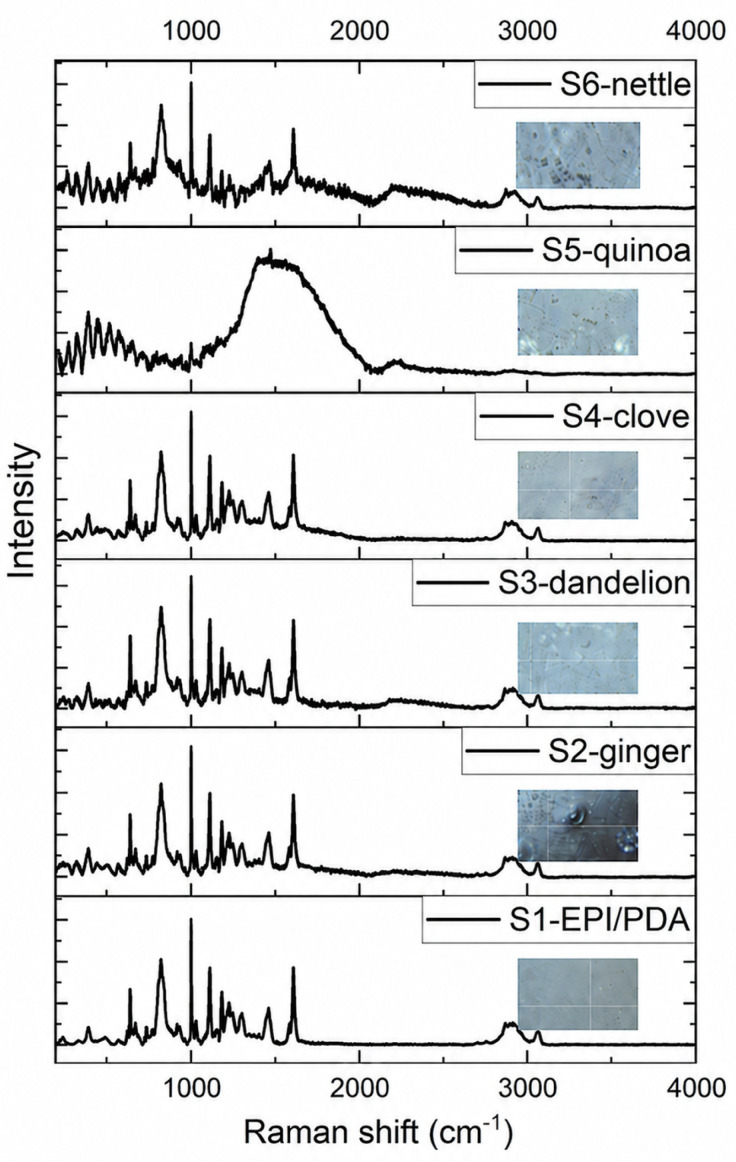
Raman spectra of EPI/PDA epoxy coatings without and with added plant-based modifiers: ginger, dandelion, clove, quinoa and stinging nettle.

The gloss of the coatings was strongly dependent on the type of modifier. For the series without UV irradiation, the highest 60° gloss value was obtained for the reference coating (121 GU). The introduction of additives generally reduced the gloss, particularly in the case of dandelion (63.8 GU) and nettle (89.3 GU). Clove and ginger, however, maintained relatively high gloss values, 109 and 113 GU, respectively, indicating better visual compatibility of these additives with the epoxy matrix. The data summarised in Table 5 and shown in Figure 6 and Figure 7 clearly indicate that the effect of modifiers on gloss was not the same for all plant additives.

The decrease in gloss should be associated with light scattering on modifier particles, local heterogeneity, and increased roughness.

The low gloss values observed for the coating containing dandelion correlate with the increased values of the roughness parameters R_a_ and R_z_. However, coatings with low gloss and higher roughness are classified as having a structural effect and are often preferred by customers, because dirt and traces of mechanical damage, such as scratches, are less visible on their surface. In contrast, in the case of coatings containing clove and ginger, despite the presence of the additive, the gloss remained relatively high, which may indicate good compatibility with the epoxy matrix, a limited effect on the surface geometry, or good dispersion of their particles in the epoxy matrix [36].

### 3.4. Raman Spectroscopy

Raman spectroscopy provides valuable information about the chemical composition and structure of the analyzed materials. This technique was used to investigate the structure of an Epidian^®^ 5 epoxy matrix cured with isophorone diamine (PDA), as well as the changes induced by natural modifiers derived from plant such as ginger, dandelion, clove, quinoa and nettle. The Raman spectra of the reference sample (Sample 1) revealed characteristic bands attributed to the cross-linked DGEBA (diglycidyl ether of bisphenol A) epoxy resin [50,51]. The most intense signals were observed at 819, 1000, 1112, and 1607 cm^−1^. These bands are assigned to O–C–O stretching vibrations, benzene ring breathing modes, C–O–C ether bridge vibrations, and C=C stretching vibrations within the bisphenol A structure, respectively [52,53,54]. Their presence confirms the characteristic structure of the DGEBA epoxy resin and indicates a high degree of crosslinking. Additional bands characteristic of residual epoxy groups were observed at 936 and 1153 cm^−1^. The low intensity of these signals suggests a high degree of oxirane group conversion during the curing process. A detailed assignment of the observed Raman bands is provided in Table 6. The Raman spectra of the EPI/PDA system serve as the reference “fingerprint” of the epoxy matrix and provide a baseline for evaluating structural changes induced by the incorporation of plant-derived modifiers. The addition of plant-based additives did not result in the appearance of new dominant bands or the disappearance of bands characteristic of the epoxy matrix. In particular, the spectra of samples ginger, clove and dandelion showed no significant differences compared with the unmodified matrix (Figure 7). The observed spectral changes were limited to slight band shifts, typically within the range of 1–5 cm^−1^ (Table 6), the appearance of some very weak additional bands with intensities only slightly exceeding the noise level, and the enhancement of selected bands in regions associated with vibrations of aromatic structures, C–O, C–C, and C–H bonds. The slight band shifts are most likely associated with the formation of additional hydrogen-bonding interactions involving functional groups present in plant-derived compounds, such as hydroxyl and methoxyl groups, as well as the interaction of these groups with hydroxyl groups formed during the epoxy curing process. The plant-derived additives used in this study contain a variety of compounds that may participate in such interactions, including cellulose, phenols, polyphenols, flavonoids, terpenoids, organic acids, chlorophyll, and other secondary metabolites [55,56,57]. These interactions may modify the properties of the polymer network of the cured epoxy resin.

Plant-derived additives contain a complex mixture of organic compounds, including polyphenols, flavonoids, terpenoids, carbohydrates (cellulose, hemicellulose), and minor amounts of organic acids and pigments such as chlorophylls and carotenoids [58]. These constituents are known to exhibit Raman-active vibrational modes [59], which may contribute to the spectral response of the modified epoxy coatings, although their detection is often hindered by low concentration and fluorescence background typical for biological materials. In particular, phenolic and flavonoid compounds are characterized by strong Raman bands associated with aromatic ring vibrations, including C=C stretching modes typically observed in the region of 1500–1650 cm^−1^, and ring breathing modes around 1000 cm^−1^ [51]. Terpenoids and essential oil components, particularly abundant in clove and ginger, may contribute to C–H stretching vibrations, as well as to C–O and C–C vibrations in the fingerprint region [59]. Polysaccharide components such as cellulose, which are expected in plant powders, are associated with Raman bands corresponding to C–O–C and C–O stretching vibrations in the range of 1000–1150 cm^−1^, as well as CH_2_ bending modes around 1450 cm^−1^ [60]. These bands overlap with the characteristic signals of the epoxy matrix, making their unambiguous identification challenging. Nevertheless, slight variations in peak intensity and minor shifts in band positions in these regions may indicate the presence of additional systems originating from plant-derived compounds.

It should also be noted that samples containing plant-derived additives exhibited an elevated spectral background, particularly pronounced in the nettle and quinoa-containing systems (Figure 7). Fluorescence is one of the most common challenges encountered in Raman spectroscopy of biological materials. In plant-derived substances, this phenomenon is primarily associated with the presence of compounds containing extended conjugated double-bond systems and chromophoric groups capable of absorbing the incident laser radiation [61]. Consequently, the fluorescence signal increases the spectral background, partially masks weak Raman bands, and reduces the signal-to-noise ratio, thereby complicating band assignment and spectral interpretation. Although this effect adversely affects spectral analysis, its occurrence is consistent with the presence of plant-derived metabolites in the modified epoxy systems.

In summary, the obtained results indicate that the plant-derived powders act not only as colorants but also influence the spectral response of the coatings. The extent of this effect depends on the type of plant material and its dispersion within the epoxy matrix. The incorporation of plant-derived additives did not interfere with the epoxy resin cross-linking process mediated by the PDA curing agent. All bands characteristic of the epoxy matrix remained present in the spectra of the modified samples. Overall, the presence of plant-based modifiers did not change the chemical structure of the coatings, which confirms only the physical nature of this modification. This observation was particularly evident for the systems containing ginger, dandelion, and clove, for which only minor spectral changes were detected. The samples containing nettle and quinoa exhibited more pronounced spectral changes, suggesting stronger interactions between the plant-derived constituents and the epoxy matrix.

### 3.5. UV-Vis-NIR Reflectance and Light Transmission

**Figure 8:**
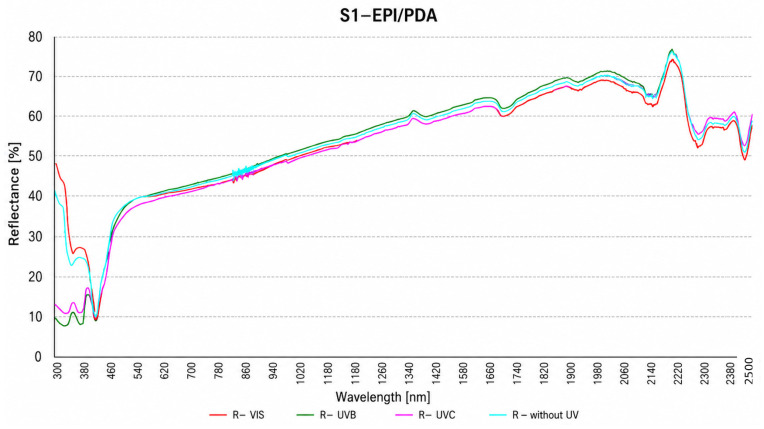
Reflectance spectrum of the reference sample S1–EPI/PDA before UV exposure and after UV-VIS, UVB, and UVC exposure.

The UV-Vis-NIR reflectance spectra showed that the greatest differences between the UV exposure series occurred in the range of 200–400 nm (UV). In the VIS-NIR region, the spectral lines were usually more parallel, which indicated a change in the level of reflectance rather than the appearance of new, strong spectral features. For some samples, UVB and UVC radiation reduced reflectance in the short-wavelength UV range, indicating modification of chromophores or of the surface layer. This result is consistent with the colour analysis, where the highest ΔE* values were observed after UVB irradiation. Figure 8 shows the reflectance R(λ) as a function of wavelength. For each composition, four curves corresponding to the following conditions are presented: without UV irradiation, UV-Vis irradiation, UVB irradiation, and UVC irradiation. In the analysis, the standard division of spectral ranges was adopted: UV (200–400 nm), VIS (400–780 nm), and NIR (780–2500 nm). For all tested samples, the spectra showed a similar character. In the range of 400–2500 nm, the reflectance spectrum changed gradually and did not show abrupt changes in the reflectance coefficient with wavelength. In contrast, in the UV region (200–400 nm), a strong decrease in reflectance to the lowest values was observed, indicating much greater absorption of radiation in this wavelength range. The differences between the irradiation variants were most visible in the range of 200–400 nm and near the UV–VIS boundary (approximately 400–500 nm), whereas for longer wavelengths, above approximately 800 nm, the reflectance curves were usually very similar. This indicates that exposure to UV radiation mainly affects the surface layer of the tested samples, while changes in optical properties at longer wavelengths are minor [62,63,64].

#### 3.5.1. Reference Sample (EPI/PDA)

The reflectance spectra correspond to pure epoxy resin without modifiers, divided into four segments: the reference sample without UV exposure and the samples subjected to UV-Vis, UVB, and UVC radiation. The reflectance spectrum for the reference sample S1–EPI/PDA is presented in Figure 8.

In the VIS–NIR range (approximately 800–2500 nm), the reflectance curves for all variants remain very similar, and the differences in reflectance level do not exceed a few percent. This indicates no significant effect of UV radiation on the optical properties of the resin in this wavelength range. The observed order of reflectance intensity (without UV ≈ UVB ≥ UV-Vis ≥ UVC) is approximate and becomes locally less distinct. Clear differences appear in the UV-VIS region, below approximately 800 nm, where the reflectance coefficient of all samples decreases sharply, reaching a minimum of approximately 10–15%. The lowest reflectance values are observed for the samples exposed to UVB and UVC radiation. Below approximately 250 nm, the reflectance coefficient of the sample without UV exposure and after UV-Vis exposure increases again to approximately 35–45%, whereas for UVB and UVC exposure it remains clearly lower. This indicates that UVB and UVC radiation lead to stronger changes in the near-surface layer of the epoxy resin than UV-Vis radiation.

#### 3.5.2. Sample with Ginger

**Figure 9:**
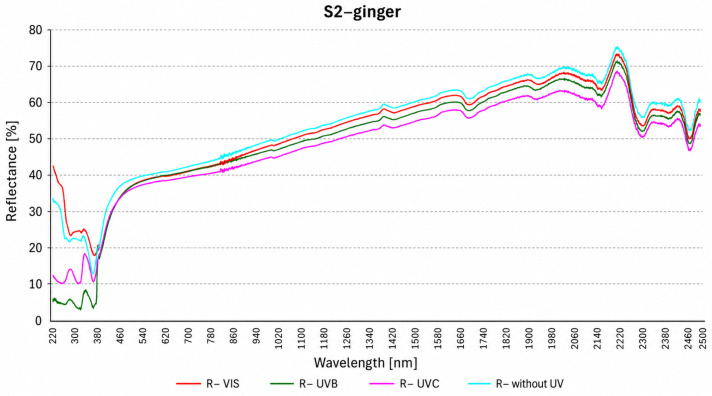
Reflectance spectra of the S2–ginger sample before UV exposure and after UV-VIS, UVB, and UVC irradiation.

In the VIS–NIR range (500–2500 nm), the reflectance curves for all irradiation variants are similar and largely parallel (Figure 9).

However, a constant tendency towards a decrease in the reflectance coefficient after exposure to UVC radiation is visible, manifested as broadband “darkening” of several percent relative to the other variants. The most characteristic differences occur in the UV region (200–400 nm). The UVB exposure variant leads to the deepest minimum of the reflectance coefficient, reaching approximately 5–10%, and to the maintenance of very low reflectance values in the deep UV. After UVC irradiation, the minimum of the reflectance curve is less pronounced and amounts to approximately 10–15%, with a similar course of the curve in the short-wavelength range. In contrast, after UV-Vis exposure, and to a lesser extent also in the absence of UV exposure, the reflectance coefficient below 250 nm increases to approximately 35–45%. This indicates the particular sensitivity of this composition to UVB radiation.

#### 3.5.3. Sample with Dandelion

**Figure 10:**
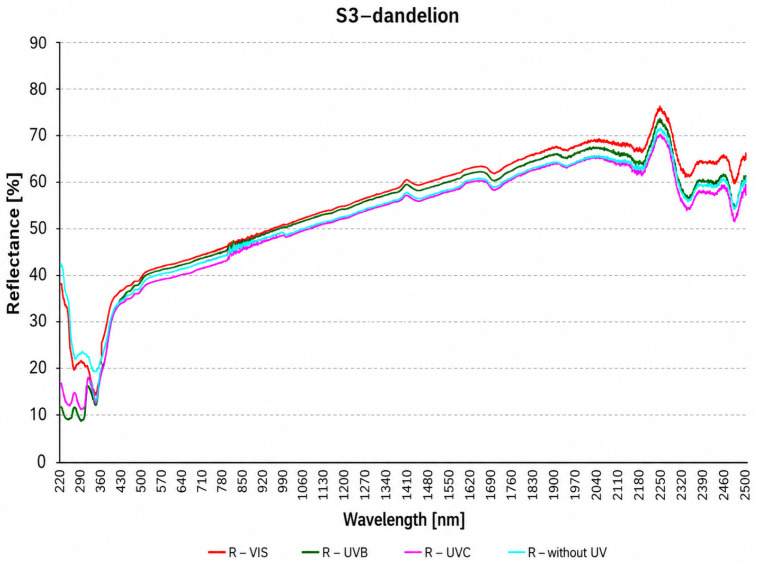
Reflectance spectra of the S3–dandelion sample before UV exposure and after UV-VIS, UVB, and UVC irradiation.

For the sample containing dandelion, in the VIS–NIR range (400–2500 nm), a shift in the reflectance level towards higher values is visible after UV-Vis exposure and, to a lesser extent, also after UVB exposure, while maintaining a similar spectral shape (Figure 10).

The differences in the reflectance coefficient level amount to several percent, indicating a change in the reflectance level rather than the appearance of new spectral features. In the UV region (200–400 nm), the reflectance coefficient decreases to approximately 10–20%. Below 250 nm, for the non-UV-exposed samples and after UV-Vis exposure, a renewed increase in the reflectance coefficient to approximately 40–45% is observed, whereas after UVB and UVC exposure the reflectance remains clearly lower. This means that UVB and UVC most strongly reduce the reflectance in the UV range, while the effect of UV-Vis radiation is clearly weaker.

#### 3.5.4. Sample with Clove

**Figure 11:**
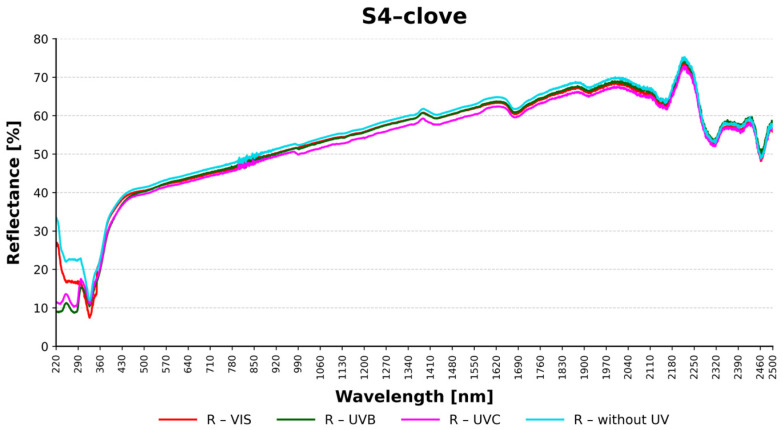
Reflectance spectra of the S4–clove sample before UV exposure and after UV-VIS, UVB, and UVC irradiation.

In the VIS–NIR range (400–2500 nm), the reflectance spectra are very similar to each other; however, the sample without UV exposure usually maintains the highest reflectance coefficient level. The reflectance spectrum of the S4–clove sample is shown in Figure 11.

Exposure to UV-Vis and UVB leads to a slight decrease in reflectance, whereas after UVC exposure the lowest reflectance values are observed, lower by several percent over the entire analysed range. In the UV region (200–400 nm), the reflectance coefficient decreases to approximately 5–15%. Below 250 nm, the reflectance values for the sample without UV exposure and after UV-Vis exposure increase to approximately 30–35%, whereas after UVB and UVC exposure they remain at a clearly lower level. The lowest reflectance coefficient values in the UV range are observed after UVB exposure, indicating its most destructive effect in this range.

#### 3.5.5. Sample with Quinoa

**Figure 12:**
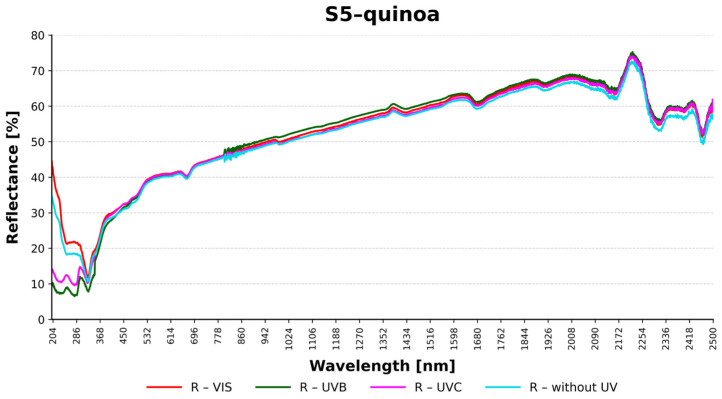
Reflectance spectra of the S5–quinoa sample before UV exposure and after UV-VIS, UVB, and UVC irradiation.

Quinoa is distinguished by the fact that, in the VIS–NIR range, exposure to UVB radiation leads to an increase in the reflectance coefficient by several percent relative to the sample without UV exposure, while maintaining a similar spectral shape. The reflectance spectrum of the S5–quinoa sample is shown in Figure 12.

After UV-Vis irradiation, this effect is weaker but still noticeable. In the UV region, the reflectance coefficient decreases to a level of several to a dozen percent. The curves after UVB and UVC exposure lie at the lowest level, whereas below 250 nm the reflectance after UV-Vis exposure increases to approximately 35–45%, with intermediate values for the sample without UV exposure. Such a divergence in the direction of changes indicates a strong dependence of the optical response of this composition on the analysed spectral range.

#### 3.5.6. Sample with Nettle

**Figure 13:**
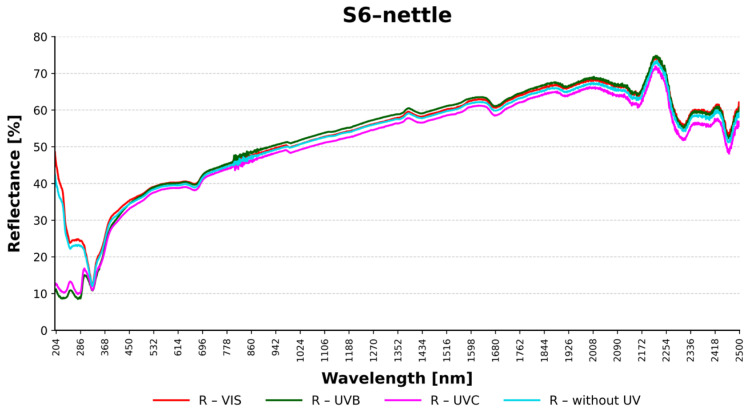
Reflectance spectra of the S6–nettle sample before UV exposure and after UV-VIS, UVB, and UVC irradiation.

In the VIS–NIR range, the highest reflectance level is observed for the sample after UVB irradiation, locally similar to that after UV-Vis irradiation, followed by the sample without UV exposure, whereas after UVC exposure the lowest reflectance coefficient values are observed, lower by several percent over the entire wavelength range. The reflectance spectrum of the S6–nettle sample is shown in Figure 13.

This means that UVC radiation most strongly reduces reflectance over a broad spectral range. In the UV region, the reflectance after UVB and UVC exposure decreases to approximately 5–15%. Below 250 nm, the reflectance coefficient for the samples without UV exposure and after UV-Vis exposure increases to approximately 35–45%, whereas after UVB and UVC exposure it remains clearly lower. As in the case of quinoa, the effect of UV radiation on the reflective properties of this composition depends on the analysed spectral window. The UV-Vis-NIR reflectance spectra show that the greatest differences between the exposure series occur in the UV range (200–400 nm). In the VIS-NIR region, the reflectance curves were usually more parallel, which mainly indicates a change in the reflectance level rather than the appearance of new, strong spectral features. For some compositions, UVB and UVC irradiation reduced reflectance in the short-wavelength UV range, which may be associated with modification of chromophores and changes in the surface layer. This result is consistent with the colour analysis, in which the highest ΔE* values were observed after UVB exposure.

### 3.6. Effective Transmittance Testing of the Resin–Substrate System Using an Electrical Method

The effective transmittance of the resin–substrate system was tested using an electrical method with a QuickSun 130CA solar radiation simulator. A Tsec4-type crystalline silicon cell was used to determine the electrical parameters. The transmittance of the epoxy resins deposited on 1.5 mm-thick commercial acrylic glass (without an anti-reflective coating) was investigated using this measurement system. The aim of the measurement was to assess the effect of UV radiation on the optical properties of epoxy resin samples containing plant modifiers. The presence of the resin sample on acrylic glass (plexi) caused a slight decrease in the effective transmittance of the tested system, determined on the basis of the current response of a silicon cell [65]. For each sample, the mean I_SC_ value was determined from four measurements performed after successive rotations of the acrylic glass by 90°, which made it possible to limit the influence of the sample position relative to the electrodes collecting the signal from the cell. The four measurements performed for different sample orientations showed very good repeatability of the results, and the relative standard deviation did not exceed approximately 0.03%. Based on the recorded operating parameters of the silicon photovoltaic cell, particularly the short-circuit current I_SC_, the attenuation of the photocurrent signal by the resin sample, ΔT, was determined, rather than the direct transmittance of the resin itself. To determine the relative transmittance of the system, T, the proportionality of the short-circuit current of the cell to the intensity of radiation reaching its surface was used. The short-circuit current of the reference silicon photovoltaic cell measured without a sample was used as the reference value for calculating the relative transmittance, (T), according to Equation (1):

(1) $$T=\frac{I_{SC}\left(plexi+resin\right)}{I_{SC}(Si\;PVcell)}.$$

The measured short-circuit current values, I_SC_, and the corresponding relative transmittance of the system, T, are presented in Table 7. The effective transmittance reduction in the entire system, ΔT, caused by the presence of the resin sample, was calculated according to Equation (2):

(2) $$∆T=T_{plexi}-T_{plexi+resin}.$$

The relative change in transmittance reduction, δT, after UV exposure with respect to the corresponding sample without UV exposure, was calculated according to Equation (3):

(3) $$\delta T=\frac{\Delta T_{\;UV\;exposure}-{\Delta T}_{without\;UV\;exposure}}{{\Delta T}_{without\;UV\;exposure}}$$

**Figure 14:**
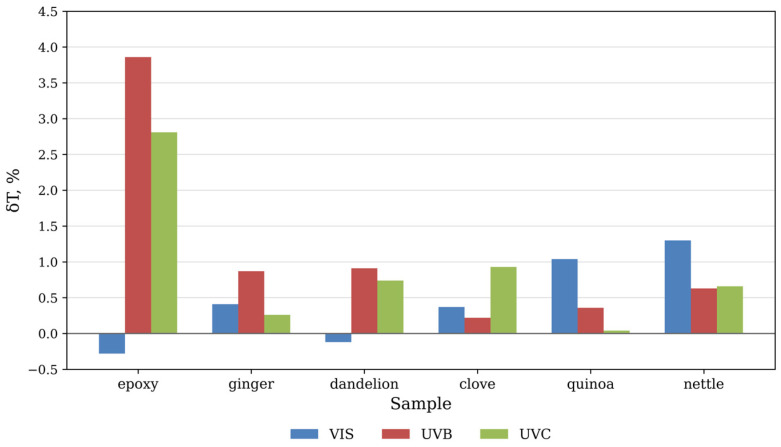
Relative change in the effective transmittance reduction δT (%) of the investigated epoxy resin samples after UV-VIS, UVB, and UVC exposure.

Negative values of δT (%) in Figure 14 indicate a greater effective reduction in transmittance after exposure to UV-VIS, UVB, or UVC radiation compared with the corresponding non-irradiated sample. This may suggest a slight increase in light absorption and/or scattering by the resin after irradiation. Although the observed changes are small, they allow comparison of the effects of UV-VIS, UVB, and UVC radiation on the optical response of the investigated resins and provide an indirect assessment of UV-induced changes in their optical properties. The calculated values of ΔT and δT are summarised in Table 7 and Table 8.

The recorded changes in δT were small but measurable and ranged from −0.28% to 3.86%. The largest increase in δT (3.86%) was observed for the reference epoxy resin sample after UVB exposure. Smaller increases were found for the samples containing ginger and dandelion modifiers. After exposure to UVC radiation, a slight increase in δT was observed for the resin containing clove pigment, whereas UV-VIS exposure produced a similar effect for the samples containing quinoa and nettle. Overall, the observed variations were minor and did not indicate a significant change in the effective transmittance of the tested resin–substrate systems. Because the measurements were performed using a crystalline silicon photovoltaic cell illuminated by the xenon lamp of the QuickSun 130CA solar simulator, the obtained results should be interpreted with respect to the overall spectral response of the measurement system rather than to a single wavelength. Consequently, the results reflect changes in the effective transmittance within the spectral range detected by the silicon photovoltaic cell, primarily the visible and near-infrared regions. Therefore, these findings should be regarded as complementary to the colour, gloss, and reflectance analyses. Short-term exposure to UV-VIS, UVB, and UVC radiation did not produce a significant change in the overall light transmittance of the investigated materials, whereas the surface and spectral characteristics—particularly the reflectance in the 200–400 nm range and the colour parameters in the CIELAB colour space—proved to be more sensitive to UV irradiation.

### 3.7. Moisture Resistance Under Condensation Conditions

**Figure 15:**
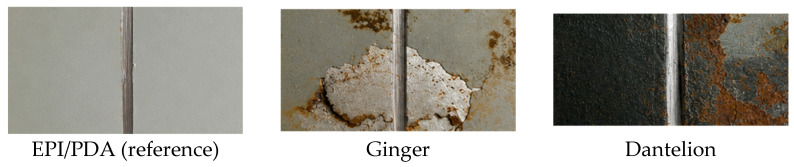

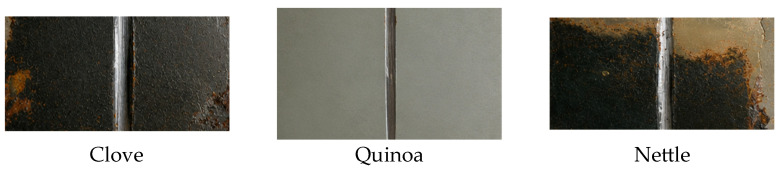
Images of coatings after 500 h of exposure in a condensation chamber.

After exposure to condensation conditions, a macroscopic assessment of the surface condition of the tested coatings was performed. The severity of the observed changes was determined using a qualitative rating scale from “−” to “+++,” where “−” indicates no visible changes, while “+++” indicates the most severe changes. The visual assessment results are summarized in Figure 15 and in Table 9.

After 500 h of exposure, the changes for the reference sample (EPI/PDA) are moderate: changes in the coating area reach a “+” level, while cracks, delamination, and subcoating corrosion are absent or only slightly visible. The coating remains continuous, confirming the relative stability of this formulation. Coatings with the addition of plant modifiers are significantly more susceptible to damage from moisture. Particularly intense changes occur in the samples with nettle, dandelion, and clove, where more discoloration, cracking, and signs of subcoating corrosion are observed. In the case of quinoa, degradation is poorly visible. Weak discoloration is present, but without significant delamination, cracking, or intense subcoating corrosion. Differences in coating resistance may result from the different microstructure of the layer after the introduction of plant-based fillers, which tend to absorb water. Moreover, lower homogeneity and structural cohesion favor local retention of condensate, which, combined with higher surface roughness (Ra/Rz), may lead to the formation of micropaths facilitating moisture penetration and accelerated degradation of the protective layer.

### 3.8. Polarized Optical Microscopy

**Figure 16:**
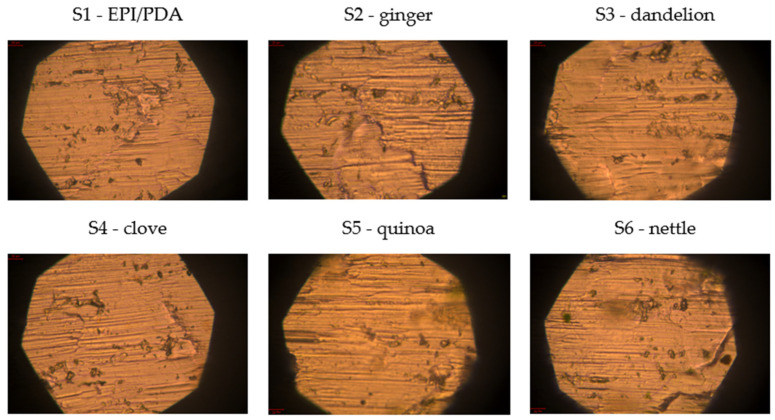
Polarized optical microscopy images of the EPI/PDA reference coating and coatings modified with ginger, dandelion, clove, quinoa, and stinging nettle.

Polarized optical microscopy was used to compare the surface morphology of the reference coating and the coatings containing plant-derived modifiers. The micrographs presented in Figure 16 reveal differences in the uniformity of the surface layer, the distribution of solid particles, and the occurrence of local defects. In all samples, parallel linear features are visible, which are associated mainly with the coating application direction and the replication of the substrate surface.

No significant differences were found between the appearance of the surfaces of the tested coatings. Thus, the optical micrographs confirm that particle size, dispersion quality, and interfacial compatibility are key parameters controlling the final performance of plant-modified epoxy coatings.

### 3.9. Physical Properties

The physical properties of the coatings were evaluated to determine how the incorporation of plant-derived modifiers affected their adhesion, hardness, scratch resistance, and frictional behaviour. These parameters provide complementary information on the interaction between the coating and the substrate, the resistance of the cured layer to local deformation, and its response to mechanical contact. The results were analysed together with the surface roughness and microscopic observations, because particle dispersion and local coating heterogeneity may directly influence the measured physical properties.

#### 3.9.1. Adhesion to Steel Substrate

**Figure 17:**
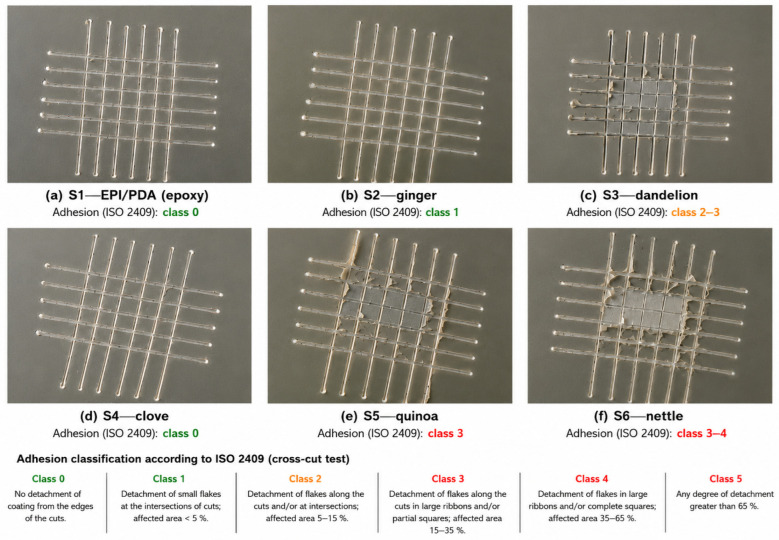
Cross-cut adhesion of the reference and plant-modified epoxy coatings on the steel substrate according to ISO 2409 [46].

The appearance of the coatings after the adhesion test is presented in Figure 16. Visual assessment of the cuts provided qualitative information on the extent of coating detachment from the substrate. To enable a direct comparison between the investigated formulations, the adhesion test results were additionally expressed using the corresponding numerical classification. The comparison presented in Figure 17 shows that the effect of the plant modifiers on adhesion depended strongly on their type and dispersion within the epoxy matrix.

The unmodified EPI/PDA coating and the coating containing clove showed the best adhesion, both being classified as class 0, with no visible detachment along the cuts. The ginger-modified coating retained very good adhesion (class 1), indicating only minor flaking at the intersections of the cuts, which is also acceptable by technical requirements. A clear deterioration was observed for dandelion (class 2–3), quinoa (class 3), and especially nettle (class 3–4). In these systems, partial coating removal occurred along the cut edges and within individual grid fields. The poorer adhesion can be attributed to local agglomeration of hydrophilic plant particles, reduced wetting by the epoxy matrix, and the formation of weak interfacial regions.

#### 3.9.2. Hardness

**Figure 18:**
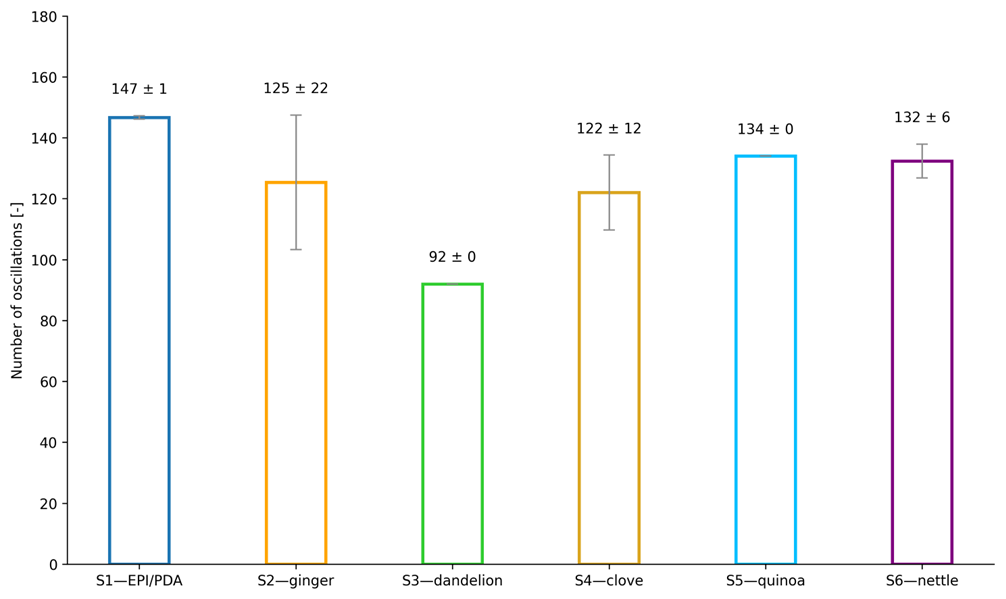
Hardness of the reference and plant-modified epoxy coatings expressed as the number of oscillations.

**Figure 19:**
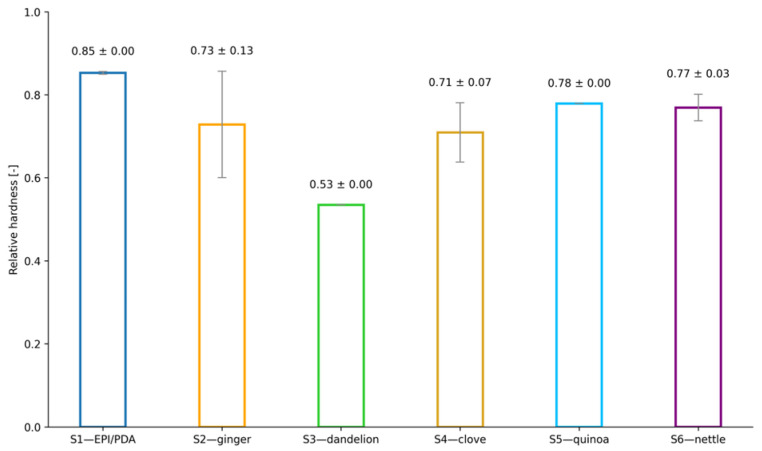
Relative hardness of the reference and plant-modified epoxy coatings.

In addition to adhesion, coating hardness was determined to assess the resistance of the cured surface to local indentation and permanent deformation. Hardness is related to the rigidity and cross-linking of the epoxy network, but it may also be affected by the presence of plant particles, weak particle–matrix interfaces, and local structural discontinuities. The hardness values obtained for the reference and plant-modified coatings are compared in Figure 18 and Figure 19.

The reference coating exhibited the highest oscillation hardness (147 ± 1), whereas the addition of all plant modifiers caused a reduction in this parameter. The strongest decrease was recorded for dandelion (92 ± 0), followed by clove (122 ± 12) and ginger (125 ± 22). Quinoa (134 ± 0) and nettle (132 ± 6) retained values closest to the reference. The same general tendency was observed for relative hardness: the reference reached 0.85 ± 0.00, while dandelion showed the lowest value of 0.53 ± 0.00. Ginger and clove reached approximately 0.73 and 0.71, respectively, whereas quinoa and nettle both reached approximately 0.78. The decrease in hardness indicates that dispersed plant particles locally disturbed the continuity and stiffness of the cross-linked epoxy network. The particularly low values obtained for dandelion correspond well with its high roughness and low gloss, confirming that this modifier produced the strongest structural disturbance of the coating surface.

#### 3.9.3. Scratch Resistance

**Figure 20:**
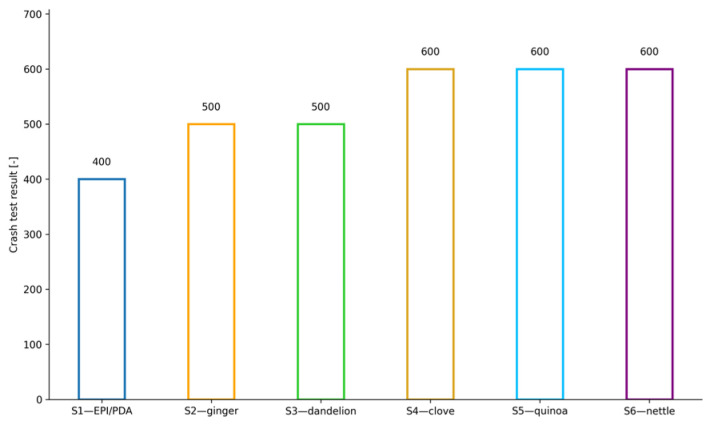
Critical load determined in the scratch-resistance test of the reference and plant-modified epoxy coatings.

Scratch resistance was evaluated separately from hardness because these parameters describe different aspects of mechanical behaviour. Whereas hardness reflects resistance to local penetration, scratch resistance additionally depends on coating cohesion, particle distribution, interfacial interactions, and the ability of the surface layer to hinder crack or groove propagation. The comparative scratch-resistance results are presented in Figure 20.

In contrast to hardness, the resistance to scratching increased after the introduction of plant-derived modifiers. The critical load increased from 400 g for the reference coating to 500 g for the ginger- and dandelion-modified coatings and to 600 g for the clove, quinoa, and nettle systems. This result suggests that solid plant particles can hinder the propagation of a scratch and increase the load required to produce a continuous visible defect. The improvement in scratch resistance despite the lower oscillation hardness indicates that these parameters describe different deformation mechanisms. Hardness reflects the overall resistance of the coating to indentation or oscillatory deformation, whereas the scratch test is additionally affected by surface texture, particle reinforcement, and friction at the moving contact.

#### 3.9.4. Coefficients of Friction

**Figure 21:**
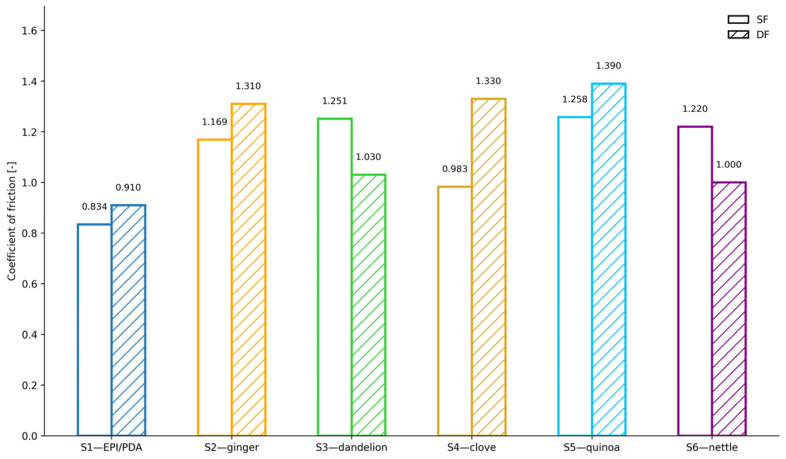
Static (SF) and dynamic (DF) coefficients of friction of the reference and plant-modified epoxy coatings.

The tribological response of the coatings was further characterised by measuring the static and dynamic coefficient of friction. These parameters are sensitive to both surface topography and the mechanical properties of the near-surface layer. Increased roughness, exposed modifier particles, and local heterogeneity may increase resistance to sliding contact, whereas a smoother and more homogeneous surface usually results in a lower coefficient of friction. The static and dynamic coefficients of friction determined for the investigated coatings are shown in Figure 21.

The reference coating exhibited the lowest friction coefficients, with SF = 0.834 and DF = 0.910. All plant modifiers increased at least one of the measured friction parameters, which can be associated with the higher roughness and more heterogeneous surface geometry of the modified coatings. For ginger and quinoa, the dynamic coefficient was higher than the static coefficient (1.310 and 1.390, respectively), indicating increased resistance during sliding. Clove showed a similar tendency, with DF = 1.330 compared with SF = 0.983. In contrast, dandelion and nettle exhibited lower dynamic than static friction coefficients. This behaviour may result from the removal or flattening of weakly bound surface asperities during sliding, leading to partial stabilization of the contact. Taken together, the friction results confirm that the plant powders alter the tribological character of the epoxy surface. The effect is governed not only by coating hardness, but also by roughness, local cohesion, and the degree of modifier–matrix bonding.

### 3.10. Structure-Property Relationship

The obtained results demonstrate that the influence of plant-derived modifiers on epoxy coatings cannot be evaluated on the basis of a single parameter. The final performance of each system resulted from the simultaneous effects of particle morphology, dispersion, interfacial compatibility with the epoxy matrix, and the chemical composition of the plant material. These factors determined the surface topography, optical response, mechanical properties, and moisture resistance.

The unmodified EPI/PDA coating exhibited the smoothest surface, the highest gloss and hardness, and very good adhesion. The incorporation of plant powders generally increased the R_a_ and R_z_ values and reduced gloss, which confirms that solid particles disturbed the continuity and uniformity of the surface layer. This relationship was particularly evident for the dandelion-modified coating, which showed the highest initial roughness, the lowest gloss and hardness, and reduced adhesion. Polarized optical microscopy revealed a homogeneous distribution of particles in the coatings, which indicates that the surface geometry and the physical properties of the cured layer are significantly influenced by the chemical structure of the modifier and the related wetting ability of the epoxy matrix.

The nettle-containing coating also exhibited pronounced surface heterogeneity, relatively high roughness, reduced adhesion, and an increased coefficient of friction. Its considerable color change after UV exposure may indicate the poor stability of the pigment contained in it, while and unfavorable behavior during the condensation test indicates the presence of weak particle–matrix interfaces, which may facilitate water penetration. Thus, the microstructural discontinuities observed in the coating were reflected not only in its mechanical response but also in its reduced humidity stability.

Quinoa produced the strongest initial color modification and the greatest color difference after UVB irradiation. The high ΔE* and Δb* values indicate that quinoa acted primarily as a natural coloring modifier, but its optical effect was accompanied by relatively high surface roughness, lower adhesion, and increased sensitivity to radiation. At the same time, the quinoa-containing coating retained comparatively high hardness and scratch resistance. This demonstrates that the effect of plant particles was not uniformly harmful: particles embedded within the matrix could locally resist penetration or scratch propagation, even when their dispersion reduced coating continuity and interfacial adhesion.

Ginger and clove provided the most balanced overall performance among the investigated modifiers. Both coatings retained relatively high gloss, while their surface roughness was lower than that of the systems containing dandelion, quinoa, or nettle. Ginger maintained good adhesion, whereas the clove-containing coating retained adhesion comparable with the reference sample. These findings suggest better wettability and compatibility of ginger and clove particles with the epoxy matrix. Nevertheless, both additives reduced coating hardness and increased the coefficient of friction, confirming that even well-dispersed plant particles modified the local surface mechanics.

All plant-modified coatings showed greater scratch resistance than the reference coating. This result may be associated with the presence of dispersed solid particles that acted as local obstacles to scratch propagation and increased the energy required for surface damage. However, scratch resistance did not correlate directly with hardness. The reduction in hardness observed for most modified systems indicates that the plant powders could locally reduce the stiffness of the cured network or create more compliant interfacial regions. Therefore, hardness, scratch resistance, adhesion, and friction should be regarded as complementary parameters describing different aspects of coating behavior.

Raman spectroscopy showed that the characteristic bands of the cured epoxy matrix remained present in all modified coatings. The absence of major new bands or disappearance of the principal epoxy signals indicates that the plant powders did not fundamentally alter the chemical structure of the cured EPI/PDA network. The observed minor band shifts, changes in intensity, and increased fluorescence background were mainly associated with the presence of plant-derived compounds and possible weak interactions, including hydrogen bonding, between the modifier constituents and the epoxy matrix. The modification was therefore primarily physical rather than based on the formation of a new polymer structure.

The optical results were consistent with the chemical and morphological observations. The greatest irradiation-induced changes in reflectance occurred in the UV range, where plant chromophores and the surface layer of the epoxy coating were most sensitive to radiation. UVB exposure produced the largest colour changes, particularly for quinoa and nettle, whereas the lower ΔE* values observed after UVC exposure may have resulted from different degradation or bleaching mechanisms. The differences between colour, reflectance, and gloss confirm that irradiation affected both the chemical composition of the chromophoric compounds and the surface morphology of the coatings.

The condensation test further demonstrated the importance of coating continuity and particle–matrix adhesion. The more heterogeneous systems, particularly those containing nettle and dandelion, showed poorer resistance to prolonged moisture exposure. Solid plant particles and agglomerates may form preferential pathways for water diffusion, while hydrophilic constituents of the plant powders may additionally promote moisture absorption. Clove retained favourable adhesion under dry conditions but showed reduced barrier performance during condensation exposure, indicating that good initial adhesion alone does not guarantee adequate long-term moisture resistance.

Overall, the results confirm that plant-derived powders can simultaneously perform several functions in solvent-free epoxy coatings. They can impart colour, modify gloss and texture, increase scratch resistance, and change frictional behaviour. However, these benefits may be accompanied by reduced hardness, adhesion, optical stability, and moisture resistance. The most favourable balance of properties was obtained for ginger and clove, whereas dandelion and nettle caused the greatest structural disturbance. Quinoa provided the strongest colouring effect but was particularly sensitive to UVB exposure.

Overall, the results confirm that plant-derived powders can simultaneously perform several functions in solvent-free epoxy coatings. They can impart color, modify gloss and texture, increase scratch resistance, and change frictional behavior. However, these benefits may be accompanied by reduced hardness, adhesion, optical stability, and moisture resistance. The most favorable balance of properties was obtained for ginger and clove, whereas dandelion and nettle caused the greatest structural disturbance. Quinoa provided the strongest coloring effect but was particularly sensitive to UVB exposure. Essentially, the key when practical use of plant powders is to ensure interfacial compatibility with the epoxy matrix, which contributes to maintaining the continuity of the coating.

## 4. Conclusions

Natural plant modifiers effectively modify the colour and appearance of epoxy coatings based on the Epidian^®^ 5/IDA system; however, their effect strongly depends on the type of raw material. The addition of modifiers increases the surface roughness compared with the reference coating; the greatest effect was obtained for dandelion, which was consistent with the reduced gloss of this coating. The greatest colour differences after UV exposure were observed after UVB irradiation, particularly for quinoa and nettle. UVB proved to be the most sensitive range for revealing optical changes in plant modifiers incorporated into epoxy resin. After UVC exposure, the ΔE* values were relatively lower, which was partly due to the yellowing of the reference sample itself and the reduced contrast between the reference and modified samples. Raman spectroscopy confirmed that the plant powders did not fundamentally alter the chemical structure of the cured epoxy matrix. The characteristic epoxy bands remained present in all samples, while minor shifts and changes in spectral background were attributed to plant-derived compounds and weak interactions with the matrix. The modification was therefore predominantly physical. UV-Vis-NIR reflectance indicated that the greatest spectral differences occurred in the UV range. Light transmission changed only slightly; therefore, this result should be treated as complementary rather than as the main research effect. The condensation test after 500 h showed that modifiers in powder form may deteriorate the barrier properties of the coatings, especially in the case of nettle, dandelion, and clove.

The physical tests showed that the modifiers generally reduced hardness but increased scratch resistance and the coefficient of friction. The higher scratch resistance of all modified coatings indicates that solid plant particles may hinder scratch propagation, even when they reduce the overall hardness of the coating. Clove retained adhesion comparable with the reference coating, while ginger also showed good adhesion. Dandelion, quinoa, and nettle produced weaker adhesion, consistent with their greater structural heterogeneity.

The obtained results indicate that natural plant modifiers may be used to design solvent-free epoxy coatings with controlled optical and decorative properties. The most promising direction for their application is safe indoor coatings, including coatings for furnishing elements, household accessories, housings, handles, decorative panels, and selected functional surfaces. Such a solution fits into the development of next-generation coating materials, in which the reduction in synthetic additives is combined with aesthetics, functionality, and concern for user health.

## Figures and Tables

**Table 1:** Composition of the investigated coating formulations.

Sample Symbol	Composition	Resin [%]	PDA [%]	Modifier [%]
S1-EPI/PDA (reference sample)	Epidian^®^ 5 + PDA	65.5	33.5	0
S2-ginger	Epidian^®^ 5 + PDA + ginger	64.5	32.5	3.0
S3-dantelion	Epidian^®^ 5 + PDA + dantelion	64.5	32.5	3.0
S4-clove	Epidian^®^ 5 + PDA + clove	64.5	32,5	3.0
S5-quinoa	Epidian^®^ 5 + PDA + quinoa	64.5	32.5	3.0
S6-nettle	Epidian^®^ 5 + PDA + nettle	64.5	32.5	3.0

**Table 2:** UV exposure conditions.

Series	Wavelength	Time
Without UV exposure	-	-
UV-VIS	460 nm	15 min
UVB	320 nm	15 min
UVC	254 nm	15 min

**Table 3:** Summary of surface roughness parameters R_a_ and R_z_ [µm], mean ± SD (n = 3).

Sample	R_a_ Without UV µm	R_a_ UV-Vis µm	R_a_ UVB µm	R_a_ UVC µm	R_z_ Without UV µm	R_z_ UV-VIS µm	R_z_ UVB µm	R_z_ UVC µm
S1–EPI/PDA	0.153 ± 0.120	0.129 ± 0.019	0.123 ± 0.031	0.137 ± 0.064	1.017 ± 0.692	0.703 ± 0.204	0.737 ± 0.058	1.003 ± 0.429
S2–ginger	0.358 ± 0.112	0.852 ± 0.551	0.345 ± 0.078	0.304 ± 0.115	1.610 ± 0.535	3.800 ± 2.523	1.507 ± 0.664	1.350 ± 0.423
S3–dandelion	2.110 ± 0.662	1.277 ± 0,177	0.537 ± 0.279	1.009 ± 0.492	8.463 ± 3.448	5.350 ± 1.001	2.487 ± 2.075	4.390 ± 3.138
S4–clove	1.031 ± 0.511	0.624 ± 0.615	0.342 ± 0.049	0.517 ± 0.480	3.807 ± 2.100	2.610 ± 2.117	1.807 ± 0.258	2.547 ± 2.342
S5–quinoa	1.044 ± 0.670	0.874 ± 0.592	1.142 ± 0.669	0.957 ± 0.521	4.117 ± 2.375	1.660 ± 0.718	4.157 ± 0.697	4.843 ± 2.835
S6–nettle	1.226 ± 0.316	0.895 ± 0.223	0.901 ± 0.346	0.905 ± 0.449	4.583 ± 1.892	3.403 ± 1.210	4.243 ± 2.301	3.747 ± 2.021

**Table 4:** Colour difference ΔE* and change in the Δb* component relative to the reference sample.

Sample	ΔE* Without UV	ΔE* UV-VIS	ΔE* UVB	ΔE* UVC	Δb* Without UV	Δb* UV-VIS	Δb* UVB	Δb* UVC
S2–ginger	0.93	2.01	3.33	2.26	−0.07	−0.39	1.92	−2.03
S3–dandelion	4.07	3.85	3.82	1.88	4.06	3.59	2.94	−1.69
S4–clove	1.00	4.06	4.25	1.84	0.94	−0.07	3.41	−1.37
S5–quinoa	6.57	6.07	7.10	0.76	6.47	5.41	6.14	0.19
S6–nettle	2.89	4.92	5.27	1.02	2.82	1.67	4.25	0.97

**Table 5:** 60° gloss [GU] of epoxy coatings.

Sample	Without UV	UV-VIS	UVB	UVC
S1–EPI/PDA	121	95.6	97.5	87
S2–ginger	113	95.7	105	97.9
S3–dandelion	63.8	62.7	80.4	65.9
S4–clove	109	103	100	103
S5–quinoa	104	95.2	81.3	85.7
S6–nettle	89.3	85.7	82.7	81.3

**Table 6:** Assignment of Raman bands and their positions in the spectra of EPI/PDA epoxy coatings without and with added plant-based modifiers.

Line Position (cm^−1^) S1—EPI/PDA	Line Position (cm^−1^) S2—Ginger	Line Position (cm^−1^) S3—Dandelion	Line Position (cm^−1^) S4—Clove	Line Position (cm^−1^) S5—Quinoa	Line Position (cm^−1^) S6—Nettle	Line Identyfication
227	230	249	248	n/a	265	skeletal vibrations
330	330	330	325	n/a	323	out-of-plane vibrations
389	393	391	391	n/a	388	arom. ring deform.
637	637	637	637	n/a	636	ν(C–C)
669	670	670	668	n/a		aromatic ring deform.
734	733	733	733	n/a	730	γ(C–H) arom.
819	822	818	820	822	818	ν(O–C–O)
914	913	912	915	911	911	ν(C–O) epoxy
936	936	934	932	940	934	>C=CH_2_, ν(C–O) epoxy
1000	1000	1000	1000	1000	1000	arom. ring breathing mode
1027	1027	1027	1028	---	1027	ν(C–O), δ(C–H)
1112	1111	1111	1111	1110	1109	ν(C–O–C), arom., δ(C-H)
1153	1154	1154	1153	1154	1146	epoxy groups
1184	1185	1182	1184	1182	1186	δ(C–H) arom.
1225	1224	1227	1223	1236	1230	ν(C–O) epoxy
1252	1253	1251	1251	---	1252	δ(C–H)
1293	1295	1296	1297	---	1293	δ(C–O–C)
1383	1380	1380	1384	1391	1386	δ(CH_2_/CH_3_)
1461	1458	1460	1457	1473	1463	δ(CH_2_)
1581	1580	1583	1581	1584	1588	ν(C=C) arom.
1607	1606	1606	1607	1609	1605	ν(C=C) arom.
2876	2869	2875	2869	2872	2873	ν(C–H)
2926	2924	2929	2906	2918	2925	ν(C–H)
3062	3065	3064	3066	3067	3062	ν(C-H) arom.

**Table 7:** Short-circuit current values, I_SC_, and the corresponding relative transmittance of the system, T, of the investigated epoxy resin samples after UV-VIS, UVB, and UVC exposure.

Sample	Isc Without UV, A	Isc UV-VIS, A	Isc UVB, A	Isc UVC, A	T Without UV	T UV-VIS	T UVB	T UVC
Tsec4 silicon cell (PV)	8.721	-	-	-	-	-	-	-
Acrylic glass	8.042	-	-	-	0.92214	-	-	-
S1–EPI/PDA	8.03884	8.03972	8.02672	8.03004	0.92178	0.92188	0.92039	0.92077
S2–ginger	8.03370	8.03030	8.02646	8.03152	0.92119	0.9208	0.92036	0.92094
S3–dandelion	8.03553	8.03631	8.02969	8.03073	0.9214	0.92149	0.92073	0.92085
S4–clove	8.03483	8.03222	8.03326	8.02820	0.92132	0.92102	0.92114	0.92056
S5–quinoa	8.03283	8.02332	8.02951	8.03248	0.92109	0.9200	0.92071	0.92105
S6–nettle	8.02620	8.02672	8.03117	8.03099	0.92033	0.92039	0.92.09	0.92088

**Table 8:** Effective transmittance reduction ΔT and relative change in transmittance reduction δT of the investigated epoxy resin samples after UV-VIS, UVB, and UVC exposure.

Sample	ΔT Without UV	ΔT UV-VIS	ΔT UVB	ΔT UVC	δT UV-VIS	δT UVB	δT UVC
S1–EPI/PDA	0.036	0.026	0.175	0.137	−0.0028	0.0386	0.0281
S2–ginger	0.095	0.134	0.178	0.120	0.0041	0.0087	0.01
S3–dandelion	0.074	0.065	0.141	0.129	−0.0012	0.0091	0.0074
S4–clove	0.082	0.112	0.100	0.158	0.0037	0.0022	0.0093
S5–quinoa	0.105	0.214	0.143	0.109	0.0104	0.0036	0.0004
S6–nettle	0.076	0.175	0.124	0.126	0.0130	0.0063	0.0066

**Table 9:** Assessment of coating degradation after 500 h of exposure in a condensation chamber.

Sample	Changes in Coating Area (Discoloration)	Cracks/ Discontinuities	Delamination/ Detachment	Signs of Underfilm Corrosion
EPI/PDA (reference)	−	−	−	−
Ginger	+	++	++	++
Dantelion	+++	++	++	+++
Clove	+++	++	++	+++
Quinoa	+/−	−	−	−
Nettle	+++	+++	+++	+++

## Data Availability

The original contributions presented in this study are included in this article. Further inquiries can be directed to the corresponding authors.
